# Efficacy and Safety of Optic Nerve Sheath Fenestration for Idiopathic Intracranial Hypertension. A Subgroup-Focused Systematic Review and Meta-Analysis

**DOI:** 10.71079/aside.im.1542545

**Published:** 2025-04-15

**Authors:** Feras Almasoud, Abduljabbar Alabduljabbar, Abdulaziz Alotaibi, Abdulbari Hanash, Yazeed Bader Alaql, Abdulwahab Alshehri, Yousef Almohammadi, Mohammad Alenazi, Abdulmajeed Alharbi, Muhammed Amir Essibayi, David J Altschul, Ahmed Y. Azzam

**Affiliations:** 1 Medical Student, College of Medicine, King Saudi University, Riyadh, Saudi Arabia.; 2 Medical Intern, King Salman Hospital, Riyadh, Saudi Arabia.; 3 Medical Student, College of Medicine, Umm Al-Qura University, Makkah, Saudi Arabia.; 4 Department of Medicine, Ministry of Health, Riyadh, Saudi Arabia.; 5 College of Medicine, Qassim University, Buraydah, Saudi Arabia.; 6 General Practitioner, King Salman Hospital, Riyadh, Saudi Arabia.; 7 Medical Intern, Eastern Health Cluster, Dammam, Saudi Arabia.; 8 Consultant of Cornea and Refractive Surgery, Department of Ophthalmology, King Salman Hospital, Riyadh, Saudi Arabia.; 9 Consultant of Ophthalmology, College of Medicine, Qassim University, Buraydah, Saudi Arabia.; 10 Montefiore-Einstein Cerebrovascular Research Lab, Albert Einstein College of Medicine, Bronx, New York, NY, USA.; 11 Department of Neurological Surgery, Montefiore Medical Center, Albert Einstein College of Medicine, Bronx, New York, NY, USA.; 12 Visiting Assistant Professor, SNU Medical Big Data Research Center, Seoul National University, Seoul, South Korea.

**Keywords:** Idiopathic Intracranial Hypertension, Optic Nerve Sheath Fenestration, Benign Intracranial Hypertension, Visual Outcomes, Systematic Review, Meta-Analysis

## Abstract

**Introduction:**

Optic nerve sheath fenestration (ONSF) is an important surgical management option for idiopathic intracranial hypertension (IIH) who failed medical treatment. We conducted a systematic review and meta-analysis to evaluate the outcomes of ONSF, with a focus to identify factors affecting treatment success.

**Methods:**

A literature search was conducted up to December 2024. Primary outcomes included improvement in visual acuity, visual fields, and optic disc swelling resolution. We performed a detailed subgroup analysis based on geographic location, study design, surgical approach, and technical variations.

**Results:**

Nineteen studies with a total of 1,159 patients were included in our study. ONSF significantly improved visual acuity in 34.5% (95% CI: 31.8–37.3%) and visual fields in 69.4% (95% CI: 65.9–72.7%) of cases. A 90.9% improvement rate was observed in reducing optic disc swelling. Significant heterogeneity was noted in visual acuity (I^2^=92.1%) and visual field improvements (I^2^=73.8%). The overall complication rate was 9% (95% CI: 5–16%). Centers that included 30 or more patients in their study demonstrated significantly lower postoperative complications.

**Conclusions:**

ONSF demonstrates favorable efficacy in improving visual outcomes with an acceptable safety profile, lower postoperative complications were observed when the procedure was performed in high-volume centers using appropriate surgical techniques. Geographic variations and surgical approaches significantly affected outcomes, highlighting the importance of standardized protocols and adequate surgical experience. Future prospective studies with standardized outcome measures are needed to optimize patient selection and surgical techniques

## 1. Introduction

Idiopathic intracranial hypertension (IIH), previously known as pseudotumor cerebri or benign intracranial hypertension, is a neurological condition characterized by elevated intracranial pressure (ICP) without identifiable structural or vascular causes [1]. This disorder mainly affects women of reproductive age and demonstrates a strong association with obesity, despite the current research about IIH, the etiopathogenesis remains not fully understood yet [1]. The hallmark clinical manifestations of the disease include headache, papilledema, and visual disturbances that may progress to blindness if left untreated with proper management [1].

The management of IIH follows a systemic approach, beginning with conservative measures such as weight loss and medical therapy, with acetazolamide, topiramate, and diuretics [2–4]. However, around 25% of patients are developing refractory IIH in which they have limited or poor response to the initial medical interventions, necessitating surgical intervention [5]. Among the available surgical options, optic nerve sheath fenestration (ONSF) is considered to be among the surgical interventions for IIH patients to preserve vision in cases of progressive visual deterioration [5, 6]. ONSF involves creating an opening in the optic nerve sheath to reduce localized cerebrospinal fluid (CSF) pressure, which by role decreases mechanical compression on the optic nerve [7]. While ONSF for IIH has been reported as a successful intervention for IIH in patients with threatened visual loss, previous studies did not have sufficient conclusive evidence about the optimal ONSF approaches and settings regarding its safety and efficacy for IIH patients [5, 8, 9].

Previous studies have highlighted important considerations in surgical procedures for IIH, but these studies either lacked complete evaluation of visual fields or have become outdated as new surgical techniques and further updated studies have been published [1, 5, 7–12]. The timing of surgical intervention appears critical, with mounting evidence suggesting that delayed intervention may result in irreversible vision loss due to progressive optic nerve damage [13]. Also, technical aspects of the procedure, such as the size and location of fenestration, muscle disinsertion requirements, and the use of minimally invasive approaches, may affect both the efficacy and complication rates of the procedure.

Recent advances in surgical techniques, especially the development of endoscopic and minimally invasive approaches, have renewed interest in evaluating the safety and efficacy of ONSF from different prospections, even in the presence of other minimally invasive interventions such as venous sinus stenting where they may not be suitable for some patients due to several factors and considerations [14]. Additionally, the emergence of large-scale studies with detailed visual outcome data has created an opportunity for a more detailed analysis of factors influencing surgical success with the option of conducting subgroup analysis to further explore more factors affecting ONSF success [15]. Therefore, we aim to evaluate the safety and efficacy of ONSF in preserving vision in patients with IIH based on the most updated evidence in the most detailed manner given the currently available data. Our study specifically focuses on three key outcomes: improvement in visual acuity, enhancement of visual fields, and resolution of optic disc swelling, in addition to investigating the reported postoperative complications from ONSF. In our study, we aim to perform detailed subgroup analyses based on geographical location, study design, surgical approach, and technical variations to identify factors that might influence surgical outcomes. Understanding these variables and factors related to ONSF in IIH patients has important considerations for optimizing patient selection and surgical technique, ultimately improving visual outcomes in this challenging patient population.

## 2. Methods:

Our systematic review and meta-analysis were conducted with adherence to the Preferred Reporting Items for Systematic Reviews and Meta-Analyses (PRISMA) guidelines. We conducted a literature search across multiple electronic databases including PubMed/MEDLINE, Embase, Web of Science, Scopus, Google Scholar, and Cochrane Library from inception through 22^nd^ of December 2024. The search strategy incorporated Medical Subject Headings (MeSH) terms and keywords related to “optic nerve sheath fenestration”, “ONSF” “idiopathic intracranial hypertension”, “IIH”, “benign intracranial hypertension”, and “pseudotumor cerebri”.

For methodological clarity, we classified all included studies into two categories: observational and interventional. It is important to note that all studies in both categories involved patients who underwent ONSF; our classification refers to study design rather than treatment allocation. Observational studies (n= 16) included retrospective chart reviews, case series, and cohort studies where the studies analyzed and reported the outcomes after ONSF without a predefined intervention protocol. Interventional studies (three studies) were those with prospective enrollment following a standardized surgical protocol and predefined outcome measures, representing a higher level of methodological confidence and validity.

### 2.1. Literature Review and Data Extraction:

Two independent reviewers screened titles and abstracts for eligibility, followed by a full-text review of the possible relevant articles. We included both observational and interventional studies reporting outcomes of ONSF in patients with IIH. Studies were eligible if they reported at least one of our primary outcomes: visual acuity improvement, visual field improvement, or optic disc swelling resolution. We excluded non-English articles, case reports or case series in which they included less than five patients, review articles, systematic reviews and meta-analyses, editorials, and letters. Data extraction was performed independently by two investigators using a standardized form. We collected information on study characteristics (publication year, country, study design), patient demographics, surgical techniques, and clinical outcomes. Complications were categorized into overall complications, diplopia, transient visual loss, worsening of visual functions, and anisocoria.

### 2.2. Risk of Bias Assessment

We assessed the risk of bias using the Risk Of Bias In Non-Randomized Studies - of Interventions (ROBINS-I) tool evaluating seven domains: confounding, selection bias, classification of interventions, deviations from intended interventions, missing data, outcome measurement, and selective reporting. The quality of evidence for each outcome was evaluated using the Grading of Recommendations, Assessment, Development, and Evaluations (GRADE) framework, considering the risk of bias, inconsistency, indirectness, imprecision, and publication bias.

### 2.3. Statistical Analysis

For statistical analysis, we performed a single-arm proportion-based meta-analysis using RStudio software with the ‘meta’ and ‘metafor’ packages which are built on R language. We calculated pooled proportions with 95% confidence intervals for each outcome using random-effects models. Heterogeneity was assessed using I^2^ statistics and Cochran’s Q test. We conducted pre-specified subgroup analyses based on country of study either, United States (US) vs. non-US based study, study type (observational vs. interventional), study design (retrospective vs. prospective), sample size (>30 vs. <30 vs. =30), surgical approach (medial transconjunctival vs. other), and surgical technique (with vs. without muscle disinsertion). Publication bias was evaluated using funnel plots Egger’s test, and the trim-and-fill method was used when appropriate. Statistical significance was set at P<0.05, and all tests were two-sided.

The rationale for our geographic subgroup analysis (US vs. non-US) was based on the differences in practice patterns, patient selection criteria, reporting standards, and healthcare systems between the regions. Previous studies on IIH have demonstrated geographic variations in disease prevalence, management approaches, and outcomes reporting [16, 17]. We aim to identify any differences in ONSF outcomes that might inform international standardization efforts and highlight region-specific considerations for optimizing patient selection and surgical technique.

## 3. Results

**Figure 1:**
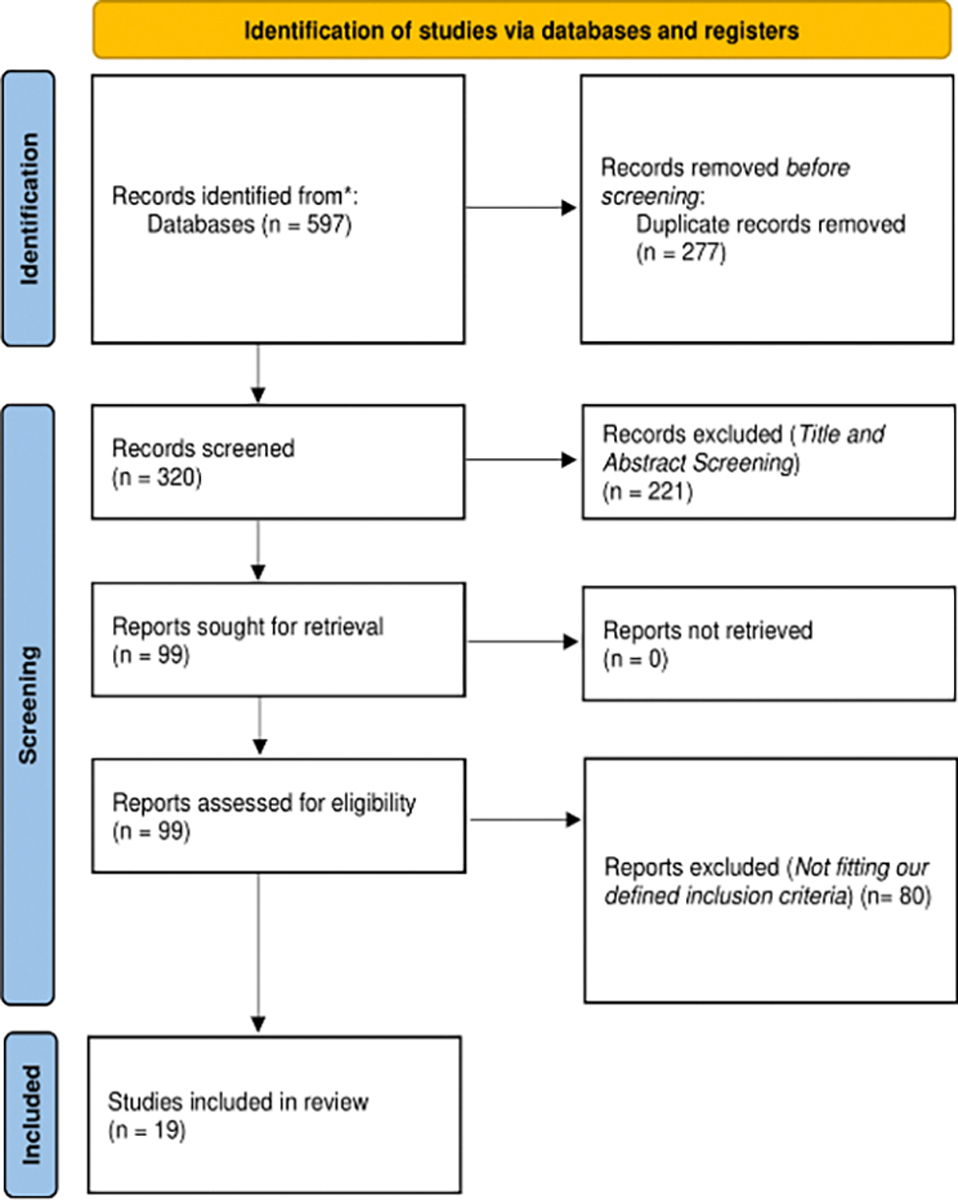
PRISMA flow chart diagram for our literature review results.

Our systematic search yielded 597 records, with 19 studies meeting the final inclusion criteria after thorough screening (Figure 1) [18–36]. The included studies spanned from 1988 to 2021, comprising 16 observational and three interventional studies, with 14 retrospective and five prospective designs (Supplementary Table 1).

### 3.1. Risk of Bias Assessment:

The ROBINS-I risk of bias assessment highlighted varied methodological quality (Supplementary Figure 1). Three studies (Melson et al., Wadikhaye et al., and Nithyanandam et al.) demonstrated a consistently low risk of bias across all domains (+). Nine studies showed serious risk of bias (X) in their overall assessment, primarily due to confounding (D1) and missing data (D5). Domain D3 (bias in classification of interventions) uniquely showed low risk (+) across all studies. Domain D4 (bias due to deviations from intended interventions) showed mixed results, with eight studies having low risk and 11 showing moderate risk. Seven studies showed consistently poor performance across multiple domains, receiving serious risk ratings in D5, D6, and D7.

### 3.2. GRADE Framework Assessment

Our GRADE framework assessment (Supplementary Table 2) revealed heterogeneous quality across outcomes. Visual acuity improvement evidence (19 studies, n=1,160) received a low-quality rating (⊕⊕○○) due to a serious risk of bias (−1) from lack of standardized visual acuity measurements and high heterogeneity (−1) with I^2^>50%. Visual field improvement (16 studies, n=719) achieved moderate quality (⊕⊕⊕○), downgraded only for serious risk bias (−1) due to varied testing methods, but showed consistent improvement across studies. Optic disc swelling resolution (11 studies, n=351) also received a moderate quality rating (⊕⊕⊕○), with consistent resolution rates and low heterogeneity.

### 3.3. Efficacy Outcomes:

Visual acuity improvement results have demonstrated statistically significant differences between non-US and US studies (48.3% vs 29.8%, p<0.001), prospective versus retrospective designs (80.8% vs 32.7%, p<0.001), and surgical approaches (medial transconjunctival 30.6% vs other 48.4%, p-value<0.001). Visual field improvement demonstrated higher rates in US versus non-US studies (72.7% vs 68.1%, p-value= 0.221) and interventional versus observational studies (83.9% vs 70.5%, p-value= 0.154). Optic disc swelling resolution showed significantly better outcomes in US studies (97.3% vs 76.5%, p-value<0.001), interventional studies (98.7% vs 83.6%, p<0.001), and medial transconjunctival approach (98.8% vs 82.8%, p-value<0.001), as listed in (Table 1). We have used the single-arm proportion-based meta-analysis as our primary analytical approach due to the nature of available data extracted from included studies. The absence of randomized controlled trials and the limited number of comparative studies precluded traditional two-arm meta-analyses using direct comparison groups and direct comparison metrics which were unavailable across most included studies.

### 3.4. Safety Outcomes

**Figure 2:**
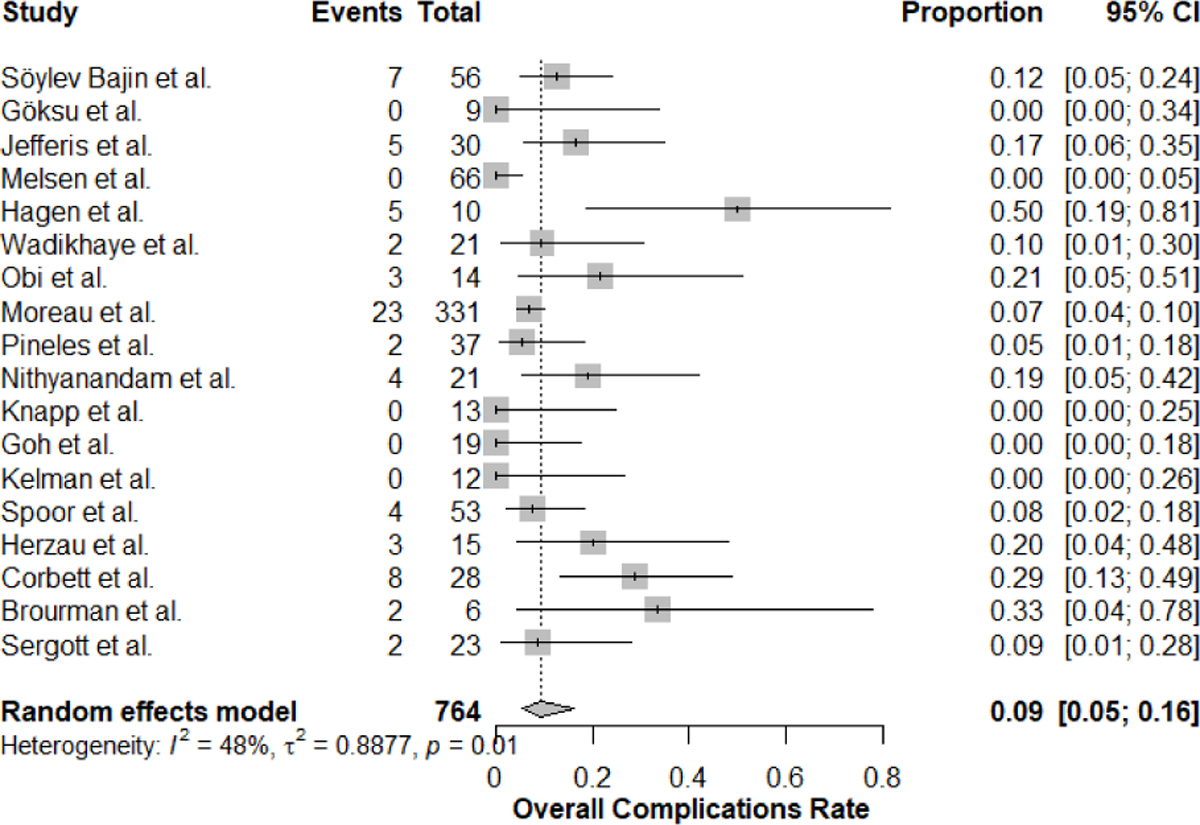
Overall rate of complications following ONSF Forest Plot.

We listed the overall reported complications from our included studies in (Table 2). The overall rate was 9% (95% CI: 5–16%, I^2^=48%), (Figure 2). Significant subgroup differences were observed in the country of study (non-US 13.9% vs US 7.4%, p-value= 0.007), surgical approach (medial transconjunctival 7.7% vs other 13.2%, p-value= 0.023), and sample size (>30: 6.6% vs <30: 15.2% vs =30: 16.7%, p-value<0.001). Specific complications showed varying rates: worsening of visual functions (8%, I^2^=51%), diplopia (3%, I^2^=24%), anisocoria (4%, I^2^=0%), and transient visual loss (10%, I^2^=69%). In addition to that, bilateral versus unilateral approaches showed significant differences in transient visual loss (6.9% vs 15.5%, p-value<0.001) and worsening of visual functions (5.7% vs 15.5%, p-value=0.018), The forest plots for the efficacy outcomes are shown in Supplementary Figure 2 **–** 5, while the subgroup analyses for safety outcomes are listed in (Supplementary Table 3).

### 3.5. Publication Bias

Publication bias assessment (Supplementary Table 4) indicated a moderate risk for visual acuity improvement (Egger’s test p-value= 0.034), with right-skewed funnel plot asymmetry and four potentially missing studies identified through trim-and-fill analysis. Visual field improvement and optic disc resolution showed low publication bias risk (Egger’s test p-value= 0.245 and p-value= 0.789, respectively).

## 4. Discussion

In our study, we evaluated the efficacy and safety outcomes of ONSF in IIH patients, focusing on detailed subgroup analyses based on multiple factors. Our findings provide important insights into the factors that may have significant considerations on ONSF outcomes and help identify optimal patient selection criteria.

Our analysis demonstrates that ONSF shows considerable efficacy in improving visual outcomes, with an overall visual acuity improvement rate of 34.5% (95% CI: 31.8–37.3%) and visual field improvement rate of 69.4% (95% CI: 65.9–72.7%). The subgroup analyses revealed several groups for efficacy and treatment success and have highlighted several important points that warrant discussion and further investigation. Studies from non-US centers showed significantly higher rates of visual acuity improvement (48.3% vs 29.8%, p-value<0.001), suggesting potential variations in patient selection criteria or surgical techniques across different geographic regions [3, 16, 37–40]. Prospective studies demonstrated markedly better outcomes in visual acuity improvement compared to retrospective designs (80.8% vs 32.7%, p-value<0.001), highlighting the importance of standardized protocols and careful patient monitoring in achieving optimal outcomes, which was previously discussed that we need more optimization and standardization for IIH studies in order to promote better quality studies and enhance our evidence about the disease [41]. The surgical approach emerged as a crucial factor, with non-medial transconjunctival approaches showing higher success rates (48.4%) compared to medial transconjunctival approaches (30.6%, p-value<0.001). This finding suggests that surgical technique selection may significantly influence visual outcomes. Sample size analysis revealed interesting patterns, with centers performing more than 30 procedures showing more consistent results compared to those with smaller case volumes. Regarding papilledema resolution, US studies demonstrated significantly better outcomes (97.3% vs 76.5%, p-value<0.001), as did interventional studies compared to observational ones (98.7% vs 83.6%, p-value<0.001), also the medial transconjunctival approach showed superior results in papilledema resolution (98.8% vs 82.8%, p-value<0.001), which is a complex and contradictory finding especially that the overall success rates were higher in non-medial transconjunctival approach as we mentioned earlier; and based on that further studies with more focused controls and approach-focused outcomes and complications should be conducted to ensure the results about it.

It is important to mention that we had statistically significant heterogeneity in visual acuity (I^2^=92.1%) and visual field improvements (I^2^=73.8%), which warrants careful interpretation. This heterogeneity likely originates from multiple sources that we have identified through additional analyses. First, patient-specific factors varied considerably across studies, with mean age ranging from 26.5 to 40.8 years, mean BMI from 26.8 to 39.6 kg/m^2^, and disease duration before ONSF from 1.3 to 36 weeks. Second, follow-up protocols differed significantly, with some studies reporting outcomes at three months and others at up to two years post-procedure, introducing temporal bias in outcome assessment. Third, the inconsistency in measurement techniques, varying from Snellen charts to log MAR for visual acuity, and from Goldmann to automated perimetry for visual fields, which created methodological variability that contributed to heterogeneous results. Also, the threshold for defining improvement was inconsistently applied across studies, with some requiring one-line improvement in visual acuity while others demanded two or more lines for positive classification.

Our safety analysis revealed an overall complication rate of 9% (95% CI: 5–16%, I^2^=48%), which is lower than previously reported in some studies from previous literature. The subgroup analyses of complications provided important significant considerations and factors to highlight risks and possible preventive strategies.

Geographic variation was significant, with non-US centers reporting higher complication rates (13.9% vs 7.4%, p=0.007). This difference might reflect variations in surgical expertise, patient selection, guidelines, or reporting practices between the US and other countries [17, 38]. The surgical approach significantly affected complication rates, with the medial transconjunctival technique showing lower complications (7.7% vs 13.2%, p=0.023), suggesting it might be the safer approach, although the surgical technique findings warrant further verification as they are contradictory within our analysis findings between different variables. Sample size-based subgroup analysis demonstrated that centers in which they performed more than 30 procedures reported in their research paper had significantly lower complication rates (6.6%) compared to those with fewer cases (15.2% for <30 cases, p<0.001), highlighting the importance of surgical experience and center volume, and may also be considered that there is a possible effect from the influence of sample size power on overall results. Specific complications showed varying patterns, with diplopia being the most common (3%, I^2^=24%), followed by anisocoria (4%, I^2^=0%).

Our findings both support and extend previous meta-analyses’ conclusions. Unlike Kalyvas et al.’s study [8], which focused broadly on various surgical interventions for IIH, our analysis provides a detailed focus on ONSF outcomes only. Compared to Friso et al.’s [42] pediatric-focused review, our study offers a comprehensive analysis across all age groups. When comparing to Santos et al. study [43], they included only ten studies, with limited outcomes assessment compared to our defined methodology and results. Also, in comparison with the recent Prokop et al. meta-analysis [44], they had several limitations in their study which we worked to overcome in our analysis including more comprehensive subgroup analysis, analysis of postoperative complications in which they did not perform, handling of publication bias using multiple statistical techniques, correction of bias through trim-and-fill technique, performing more detailed risk of bias assessment, in addition to the introduction of GRADE framework approach to our analysis in which they did not perform.

Despite our given analysis strengths and novel points compared to previous studies, our study has several limitations that warrant acknowledgment. First, the retrospective nature of most included studies introduces selection and reporting biases. Second, the heterogeneity in outcome reporting and surgical techniques across studies may affect the generalizability of our findings. Third, the lack of standardized visual outcome measurements across studies made some comparisons challenging. Future studies should focus on prospective data collection with standardized outcome measures and longer follow-up periods. Multicenter randomized controlled trials comparing different surgical approaches would be valuable in definitively establishing the optimal technique. Additionally, studies investigating the role of modern surgical adjuncts and their impact on outcomes would be beneficial.

## 5. Conclusion

ONSF demonstrated significant efficacy in improving visual outcomes, with promising results in visual field improvement (69.4%) and papilledema resolution (90.9%). The procedure’s effectiveness varied between different settings and approaches, with prospective studies and non-medial transconjunctival approaches showing superior visual acuity improvement rates. Centers performing more than 30 procedures demonstrated better outcomes and lower complication rates, suggesting a volume-outcome relationship in ONSF procedures. The overall safety profile was favorable, with a 9% complication rate, mostly including manageable complications such as diplopia (3%) and anisocoria (4%). The medial transconjunctival approach emerged as the safer technique with significantly lower complication rates (7.7% vs 13.2%), despite that non-medial transconjunctival approaches demonstrated better efficacy outcomes earlier. Geographic variations in both efficacy and safety outcomes address the importance of standardizing surgical techniques and patient selection criteria. US centers showed better papilledema resolution rates and better safety profiles compared to non-US centers, suggesting possible differences in practice patterns that warrant further investigation. These findings support ONSF as a viable surgical option for IIH patients, especially when performed in experienced centers using appropriate surgical techniques. Future studies should focus on prospective studies with standardized outcome measures and surgical protocols to further optimize patient outcomes. Also, the development of formal training programs and surgical guidelines could help reduce the observed variations in outcomes across different centers and regions.

## Supplementary Material

Supplementary Files

## Figures and Tables

**Table 1:** Meta-Analysis of ONSF Outcomes and Subgroup Analyses

Outcome/Subgroup Category	Subgroup	Events/Total	Proportion (95% CI)	P-value

Visual Acuity Improvement	Overall	400/1160	0.345 (0.318–0.373)	**<0.001** *
Country	Non-US	153/317	0.483 (0.428–0.538)	**<0.001** *
	US	243/816	0.298 (0.267–0.330)	
Study Type	Observational	312/931	0.335 (0.306–0.366)	**0.034** *
	Interventional	84/202	0.416 (0.350–0.485)	
Study Design	Retrospective	354/1081	0.327 (0.300–0.356)	**<0.001** *
	Prospective	42/52	0.808 (0.681–0.892)	
Surgical Approach	Medial Transconjunctival	261/854	0.306 (0.276–0.337)	**<0.001** *
	Other	135/279	0.484 (0.426–0.542)	
Sample Size	>30	242/800	0.302 (0.272–0.335)	**<0.001** *
	<30	128/277	0.462 (0.404–0.521)	
	=30	26/56	0.464 (0.340–0.593)	
Visual Field Improvement	Overall	499/719	0.694 (0.659–0.727)	**<0.001** *
Country	Non-US	169/248	0.681 (0.621–0.736)	0.221
	US	323/444	0.727 (0.684–0.767)	
Study Type	Observational	466/661	0.705 (0.669–0.738)	0.154
	Interventional	26/31	0.839 (0.674–0.929)	
Study Design	Retrospective	466/661	0.705 (0.669–0.738)	0.154
	Prospective	26/31	0.839 (0.674–0.929)	
Surgical Approach	Medial Transconjunctival	353/496	0.712 (0.670–0.750)	0.999
	Other	139/196	0.709 (0.642–0.768)	
Sample Size	>30	312/436	0.716 (0.672–0.756)	0.930
	<30	143/204	0.701 (0.635–0.760)	
	=30	37/52	0.712 (0.577–0.817)	
Optic Disc Resolution	Overall	319/351	0.909 (0.874–0.935)	**<0.001** *
Country	US	220/226	0.973 (0.943–0.988)	**<0.001** *
	Non-US	75/98	0.765 (0.672–0.838)	
Study Type	Interventional	157/159	0.987 (0.955–0.997)	**<0.001** *

ONSF: Optic Nerve Sheath Fenestration; CI: Confidence Interval; US: United States.

* Denotes Statistical Significance; P-values represent a comparison between subgroups within each category; Overall results for each outcome represent the pooled analysis across all studies

**Table 2:** Postoperative Complications Rate

Study	Diplopia	Transient visual loss	Worsening of visual functions	Anisocoria	Overall complications
Söylev Bajin et al.[18]	NR	21/81	11/112	NR	7/56
Göksu et al. [19]	NR	0/9	0/9	NR	0/9
Jefferis et al. [20]	0/30	2/30	2/30	NR	5/30
Melson et al. [21]	0/66	4/66	4/66	NR	0/66
Hagen et al. [22]	NR	NR	NR	NR	5/10
Wadikhaye et al. [23]	NR	0/21	0/21	2/21	2/21
Obi et al. [24]	4/14	4/14	4/14	NR	3/14
Moreau et al. [25]	20/331	32/568	32/568	NR	23/331
Pineles et al. [26]	2/37	9/37	9/37	2/37	2/37
Nithyanandam et al. [27]	NR	2/21	2/41	NR	4/21
Knapp et al. [28]	NR	4/27	4/27	NR	0/13
Goh et al. [29]	0/19	0/19	0/19	0/19	0/19
Acheson et al. [30]	NR	3/14	3/14	NR	NR
Kelman et al. [31]	0/12	0/12	0/12	0/12	0/12
Spoor et al. [32]	NR	NR	NR	NR	4/53
Herzau et al. [33]	NR	3/15	3/27	NR	3/15
Corbett et al. [34]	NR	5/40	5/NR	NR	8/28
Brourman et al. [35]	1/6	1/6	1/6	NR	2/6
Sergott et al. [36]	NR	NR	NR	NR	2/23

NR: Not Reported; ONSF: Optic Nerve Sheath Fenestration

## Data Availability

This review article does not contain any new primary data. All information discussed is derived from previously published sources and publicly available databases, as cited in the manuscript.
