# Supplementary material for: Efficacy and Safety of Optic Nerve Sheath Fenestration for Idiopathic Intracranial Hypertension. A Subgroup-Focused Systematic Review and Meta-Analysis
Source: ASIDE Intern Med. Author manuscript; Available in PMC 2025 Jul 22. (PMC12282507; doi:10.71079/aside.im.1542545)
Supplement: Supplementary Files [file NIHMS2075468-supplement-Supplementary_Files.docx]

**Contents**

[**Figures:** 2](#_Toc195708279)

[**Supplementary Figure 1: Risk of Bias Assessment (ROBINS-I).** 2](#_Toc195708280)

[**Supplementary Figure 2: Rate of Diplopia Forest Plot.** 3](#_Toc195708281)

[**Supplementary Figure 3: Rate of Anisocoria Forest Plot.** 4](#_Toc195708282)

[**Supplementary Figure 4: Rate of Transient Visual Loss Forest Plot.** 5](#_Toc195708283)

[**Supplementary Figure 5: Rate of Worsening of Visual Function Forest Plot.** 6](#_Toc195708284)

[**Tables Legend:** 7](#_Toc195708285)

[**Supplementary Table 1: Baseline Characteristics of The Included Studies.** 7](#_Toc195708286)

[**Supplementary Table 2: GRADE Framework Assessment of ONSF Outcomes.** 9](#_Toc195708287)

[**Supplementary Table 3: Subgroup Analysis of ONSF Complications.** 10](#_Toc195708288)

[**Supplementary Table 4: Publication Bias Assessment of Outcomes.** 12](#_Toc195708289)

# **Figures:**

## **Supplementary Figure 1: Risk of Bias Assessment (ROBINS-I).**


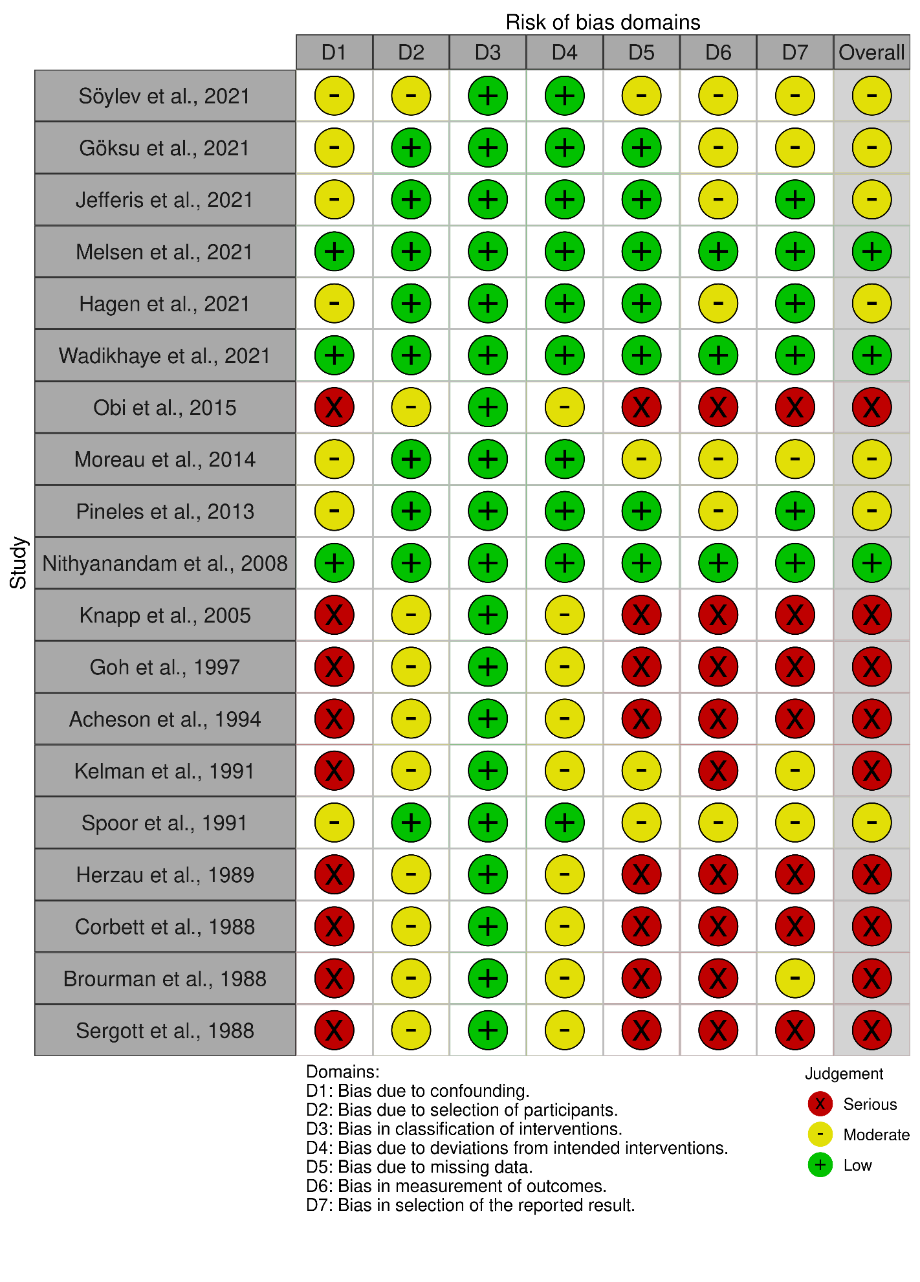


## **Supplementary Figure 2: Rate of Diplopia Forest Plot.**


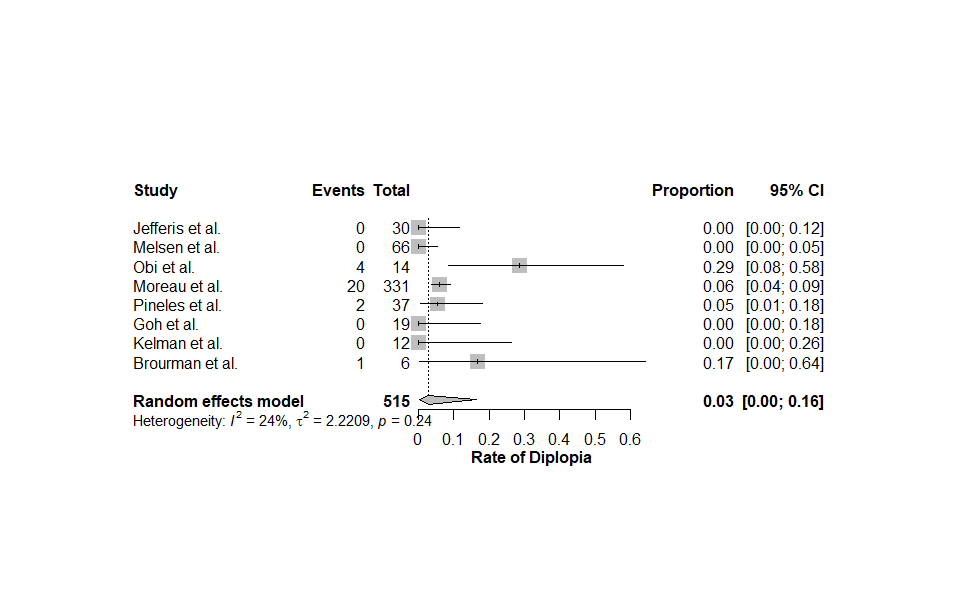


## **Supplementary Figure 3: Rate of Anisocoria Forest Plot.**


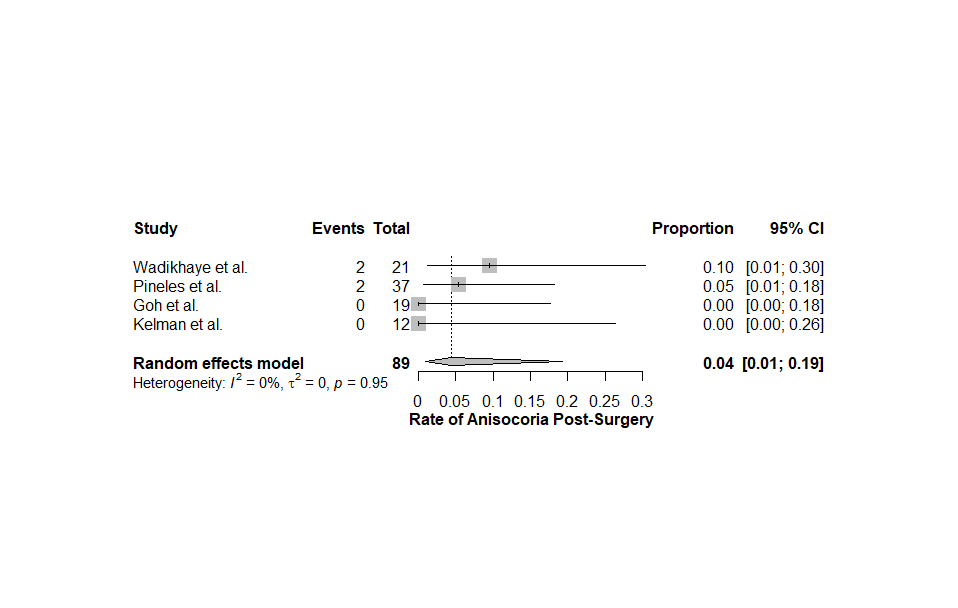


## **Supplementary Figure 4: Rate of Transient Visual Loss Forest Plot.**


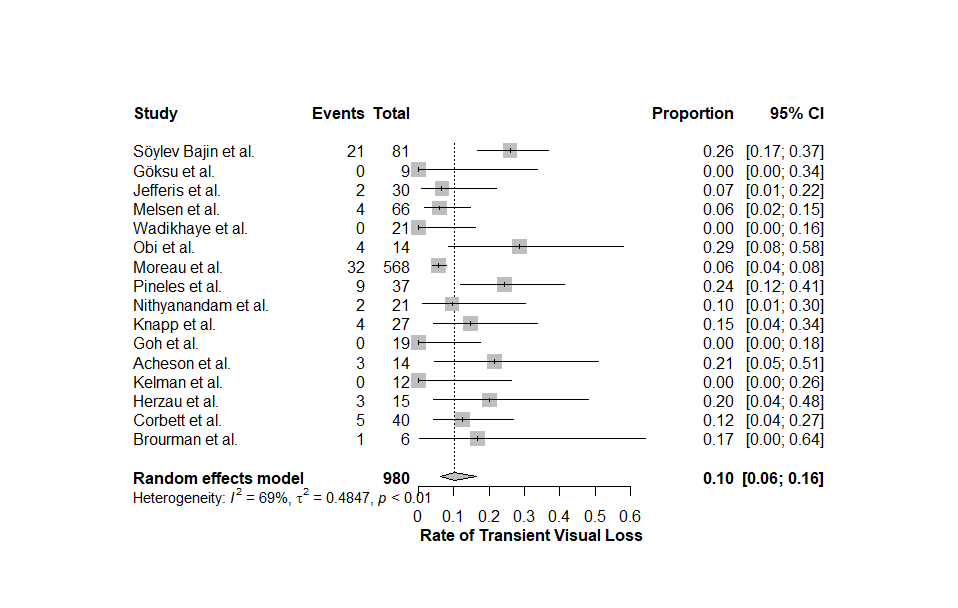


## **Supplementary Figure 5: Rate of Worsening of Visual Function Forest Plot.**

**
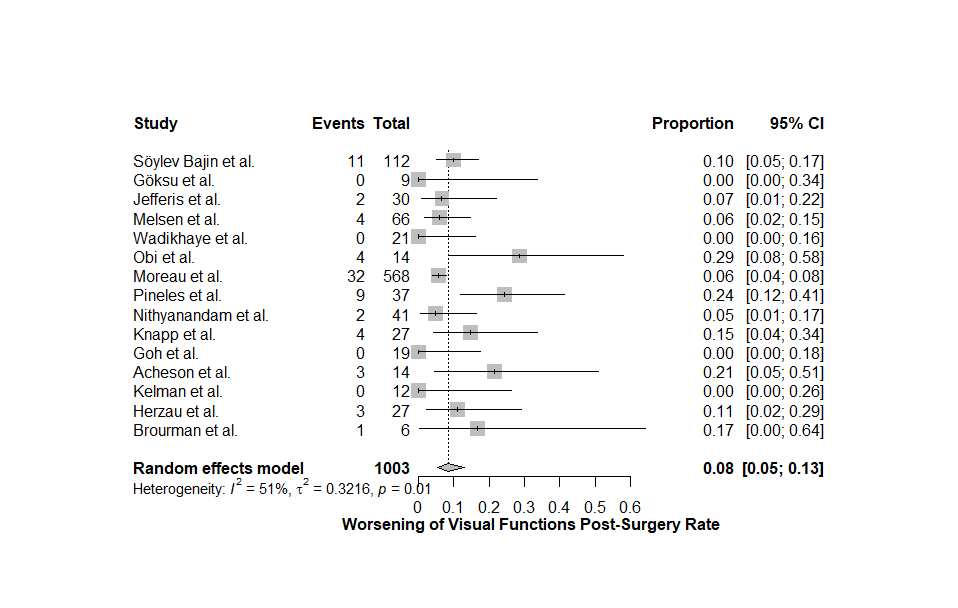
**

# **Tables Legend:**

## **Supplementary Table 1: Baseline Characteristics of The Included Studies.**

| **Study** | **Publication Year** | **Country of Study** | **Study Type** | **Study Design** | **Surgical Approach** | **Number of Patients** | **Instrumentation** | **Muscle Disinsertion** | **Mean Age (Years), SD** | **Mean BMI, SD** | **Mean LP CSF Opening Pressure (mm H2O), SD** | **Previous Surgical Interventions, Percentage** | **ONSF Approach** | **Mean Time to Surgery (Weeks), SD** | **Dominant Approach** | **Unilateral, Percentage** | **Bilateral, Percentage** |
| --- | --- | --- | --- | --- | --- | --- | --- | --- | --- | --- | --- | --- | --- | --- | --- | --- | --- |
| Söylev Bajin et al. [26] | 2021 | Turkey | Observational | Retrospective cohort study | Medial transconjunctival | 56 | Non-specified | Yes | 32.8 (8.7) F, 44.5 (8.6) M | N/A | 400 (162) | N/A | Both | 10.42, 16 | No dominant approach | 55% | 45% |
| Göksu et al. [27] | 2021 | Turkey | Interventional | Retrospective case series | Transnasal endoscopic | 9 | Endoscope | No | 40.8 (N/A) | N/A | N/A | 11.10% | Bilateral | N/A | Bilateral | 0% | 100% |
| Jefferis et al. [28] | 2021 | UK | Observational | Retrospective analysis | Supero-medial eyelid skin crease approach | 30 | Microscope | No | 30.4 (9.6) | 39.6 (7.9) | N/A | 10% | Bilateral | N/A | Bilateral | 10% | 83.30% |
| Melson et al. [29] | 2021 | USA | Interventional | Retrospective case series | Medial transconjunctival | 66 | Microscope | No | 30 (N/A) | 36 (N/A) | 380 (N/A) | N/A | Bilateral | 16, N/A | Bilateral | 0% | 100% |
| Hagen et al. [30] | 2021 | Denmark | Observational | Retrospective chart review | Superonasal transconjunctival | 10 | Microscope | No | 28.7 (11.4) | 34.4 (4.8) | 503 (120) | 0% | Unilateral | 1.3, N/A | Unilateral | 100% | 0% |
| Wadhkhaye et al. [31] | 2021 | India | Interventional | Prospective cohort study | Fronto-temporo-sphenoidotomy | 21 | Microscope | No | 27.47 (N/A) | 26.80 (N/A) | 389 (N/A) | 19.04% | Unilateral | N/A | Unilateral | 100% | 0% |
| Obi et al. [32] | 2015 | UK | Observational | Retrospective cohort study | Medial transconjunctival | 14 | Non-specified | Yes | N/A | N/A | N/A | N/A | Both | N/A | N/A | N/A | N/A |
| Moreau et al. [33] | 2014 | USA | Observational | Retrospective review | Medial transconjunctival | 236 | Non-specified | Yes | N/A | N/A | N/A | N/A | Medial transconjunctival | N/A | N/A | N/A | N/A |
| Pineles et al. [34] | 2013 | USA | Observational | Retrospective record review | Non-specified | 37 | Non-specified | N/A | 33 (11) | N/A | >250 (N/A) | 0% | Both | 36, 54 | Unilateral | 64.90% | 35.10% |
| Nithyanandam et al. [35] | 2008 | India | Interventional | Prospective noncomparative | Medial transconjunctival | 5 | Microscope | Yes | 29.5 (8.2) | N/A | >250 (N/A) | N/A | Bilateral | N/A | Bilateral | 40% | 60% |
| Knapp et al. [36] | 2005 | UK | Observational | Retrospective case series | Medial transconjunctival | 13 | Non-specified | Yes | 26.5 (N/A) | N/A | N/A | 7.70% | Both | N/A | N/A | N/A | N/A |
| Goh et al. [37] | 1997 | Singapore/USA | Observational | Retrospective case series | Medial and lateral orbitotomies | 19 | Non-specified | N/A | 33.1 (N/A) | N/A | N/A | N/A | Both | N/A | Bilateral | 47.40% | 52.60% |
| Acheson et al. [38] | 1994 | UK | Observational | Retrospective review | Medial transconjunctival | 11 | Microscope | Yes | 37 (N/A) | N/A | >250 (N/A) | 28.60% | Both | N/A | No dominant approach | 57.10% | 42.90% |
| Kelman et al. [39] | 1993 | USA | Observational | Retrospective case series | Posterolateral transconjunctival | 12 | Microscope | Yes | N/A | N/A | >240 (N/A) | 26.10% | N/A | Initially unilateral | N/A | Unilateral | 71.40% |
| Spoor et al. [40] | 1991 | USA | Observational | Retrospective review | Medial transconjunctival | 53 | Microscope | Yes | 32.5 (N/A) | N/A | N/A | N/A | Transconjunctival medial | N/A | N/A | N/A | N/A |
| Herzau et al. [41] | 1989 | Germany | Observational | Retrospective case analysis | Non-specified | 15 | Non-specified | N/A | 34 (N/A) | N/A | N/A | N/A | Both | N/A | Bilateral | 20% | 80% |
| Corbett et al. [42] | 1988 | USA | Observational | Retrospective case series | Lateral or combined lateral and medial orbitotomy | 28 | Non-specified | N/A | 29.5 (N/A) | N/A | >250 (N/A) | 3.60% | Both | N/A | N/A | N/A | N/A |
| Brourman et al. [43] | 1988 | USA | Observational | Retrospective case series | Medial transconjunctival | 6 | Non-specified | Yes | N/A | N/A | >300 (N/A) | 16.70% | Both | N/A | No dominant approach | 50% | 50% |
| Sergott et al. [44] | 1988 | USA | Observational | Retrospective case series | Posterior transconjunctival | 23 | Microscope | Yes | 38.1 (N/A) | N/A | >240 (N/A) | 26.10% | Initially unilateral | N/A | Unilateral | 71.40% | 28.60% |

*SD: Standard Deviation; F: Female; M: Male; BMI: Body Mass Index; LP: Lumbar Puncture; CSF: Cerebrospinal Fluid; ONSF: Optic Nerve Sheath Fenestration; N/A: Not Available or Not Applicable; US: United States; UK: United Kingdom; mm H₂O: millimeters of water.*

## **Supplementary Table 2: GRADE Framework Assessment of ONSF Outcomes.**

| Outcome | Study Design | Risk of Bias | Inconsistency | Indirectness | Imprecision | Publication Bias | Other Considerations | Quality of Evidence |
| --- | --- | --- | --- | --- | --- | --- | --- | --- |
| Visual Acuity Improvement (19 studies, n=1160) | Mostly observational studies (16/19) | Serious (-1) • Lack of standardized VA measurement • Retrospective design in most studies | Serious (-1) • High heterogeneity (I²>50%) • Variable effect sizes across studies | Not serious | Not serious • Large sample size • Narrow confidence intervals | Not suspected | • Strong effect in prospective studies • Dose-response gradient observed | LOW ⊕⊕○○ |
| Visual Field Improvement (16 studies, n=719) | Mostly observational studies (14/16) | Serious (-1) • Varied VF testing methods • Mostly retrospective studies | Not serious • Consistent improvement across studies • Moderate heterogeneity | Not serious | Not serious • Adequate sample size • Precise estimates | Not suspected | • Consistent effect across subgroups • Large magnitude of effect | MODERATE ⊕⊕⊕○ |
| Optic Disc Resolution (11 studies, n=351) | Mixed design (8 observational, 3 interventional) | Serious (-1) • Variable follow-up periods • Subjective assessment in some studies | Not serious • Consistent resolution rates • Low heterogeneity | Not serious | Not serious • High event rates • Narrow confidence intervals | Not suspected | • Very large effect size • Consistent across approaches | MODERATE ⊕⊕⊕○ |

**GRADE Assessment Criteria:**
• Risk of Bias: Limitations in study design or execution
• Inconsistency: Unexplained heterogeneity in results
• Indirectness: Differences in population, intervention, or outcomes
• Imprecision: Wide confidence intervals or small sample size
• Publication Bias: Systematic under/over-estimation of effect

**Quality of Evidence Ratings:**
• HIGH (⊕⊕⊕⊕): Further research very unlikely to change confidence in effect estimate
• MODERATE (⊕⊕⊕○): Further research likely to impact confidence in effect estimate
• LOW (⊕⊕○○): Further research very likely to impact confidence in effect estimate
• VERY LOW (⊕○○○): Very uncertain about the effect estimate

**Notes:**
1. -1 indicates downgrade by one level for that criterion
2. VA = Visual Acuity; VF = Visual Field
3. Sample sizes (n) represent total patients across all studies for each outcome

**Abbreviations**:

GRADE= Grading of Recommendations, Assessment, Development and Evaluations; ONSF= Optic Nerve Sheath Fenestration.

## **Supplementary Table 3: Subgroup Analysis of ONSF Complications.**

| **Complication Type** | **Subgroup Category** | **Subgroup** | **Events/Total** | **Proportion (95% CI)** | **P-value** |
| --- | --- | --- | --- | --- | --- |
| **Overall Complications** | Country | Non-US | 29/208 | 0.139 (0.099-0.193) | **0.007*** |
|  |  | US | 41/556 | 0.074 (0.055-0.099) |  |
|  | Study Type | Observational | 64/647 | 0.099 (0.078-0.124) | 0.117 |
|  |  | Interventional | 6/117 | 0.051 (0.024-0.107) |  |
|  | Study Design | Retrospective | 64/722 | 0.089 (0.070-0.112) | 0.264 |
|  |  | Prospective | 6/42 | 0.143 (0.067-0.278) |  |
|  | Surgical Approach | Medial Transconjunctival | 43/560 | 0.077 (0.058-0.102) | **0.023*** |
|  |  | Other | 27/204 | 0.132 (0.093-0.186) |  |
|  | Sample Size | >30 | 36/543 | 0.066 (0.048-0.090) | **<0.001*** |
|  |  | <30 | 29/191 | 0.152 (0.108-0.210) |  |
|  |  | =30 | 5/30 | 0.167 (0.073-0.336) |  |
|  | Muscle Disinsertion | Yes | 45/529 | 0.085 (0.064-0.112) | 0.344 |
|  |  | No | 25/235 | 0.106 (0.073-0.152) |  |
|  | Dominant Approach | Bilateral | 12/160 | 0.075 (0.043-0.127) | 0.273 |
|  |  | Unilateral | 11/91 | 0.121 (0.069-0.204) |  |
| **Diplopia** | Country | Non-US | 4/63 | 0.063 (0.025-0.152) | 0.559 |
|  |  | US | 23/452 | 0.051 (0.034-0.075) |  |
|  | Study Type | Observational | 27/449 | 0.060 (0.042-0.086) | **0.036*** |
|  |  | Interventional | 0/66 | 0.000 (0.000-0.055) |  |
|  | Surgical Approach | Medial Transconjunctival | 25/417 | 0.060 (0.041-0.087) | 0.135 |
|  |  | Other | 2/98 | 0.020 (0.006-0.071) |  |
|  | Sample Size | >30 | 22/434 | 0.051 (0.034-0.076) | 0.148 |
|  |  | <30 | 5/51 | 0.098 (0.043-0.210) |  |
|  |  | =30 | 0/30 | 0.000 (0.000-0.114) |  |
|  | Muscle Disinsertion | Yes | 25/363 | 0.069 (0.047-0.100) | **0.008*** |
|  |  | No | 2/152 | 0.013 (0.004-0.047) |  |
| **Transient Visual Loss** | Country | Non-US | 39/251 | 0.155 (0.116-0.205) | **<0.001*** |
|  |  | US | 51/729 | 0.070 (0.054-0.091) |  |
|  | Study Type | Observational | 84/863 | 0.097 (0.079-0.119) | 0.124 |
|  |  | Interventional | 6/117 | 0.051 (0.024-0.107) |  |
|  | Study Design | Retrospective | 88/938 | 0.094 (0.077-0.114) | 0.420 |
|  |  | Prospective | 2/42 | 0.048 (0.013-0.158) |  |
|  | Surgical Approach | Medial Transconjunctival | 71/797 | 0.089 (0.071-0.111) | 0.570 |
|  |  | Other | 19/183 | 0.104 (0.067-0.156) |  |
|  | Sample Size | >30 | 66/752 | 0.088 (0.070-0.110) | 0.533 |
|  |  | <30 | 22/198 | 0.111 (0.075-0.162) |  |
|  |  | =30 | 2/30 | 0.067 (0.018-0.213) |  |
|  | Muscle Disinsertion | Yes | 67/743 | 0.090 (0.072-0.113) | 0.796 |
|  |  | No | 23/237 | 0.097 (0.066-0.141) |  |
|  | Dominant Approach | Bilateral | 11/160 | 0.069 (0.039-0.119) | **<0.001*** |
|  |  | Unilateral | 9/58 | 0.155 (0.084-0.269) |  |
| **Worsening of Visual Functions** | Country | Non-US | 29/314 | 0.092 (0.065-0.129) | 0.156 |
|  |  | US | 46/689 | 0.067 (0.050-0.088) |  |
|  | Study Type | Observational | 69/866 | 0.080 (0.063-0.100) | 0.163 |
|  |  | Interventional | 6/137 | 0.044 (0.020-0.092) |  |
|  | Study Design | Retrospective | 73/941 | 0.078 (0.062-0.096) | 0.313 |
|  |  | Prospective | 2/62 | 0.032 (0.009-0.110) |  |
|  | Surgical Approach | Medial Transconjunctival | 61/848 | 0.072 (0.056-0.091) | 0.408 |
|  |  | Other | 14/155 | 0.090 (0.055-0.146) |  |
|  | Sample Size | >30 | 56/783 | 0.072 (0.055-0.092) | 0.690 |
|  |  | <30 | 17/190 | 0.089 (0.057-0.139) |  |
|  |  | =30 | 2/30 | 0.067 (0.018-0.213) |  |
|  | Muscle Disinsertion | Yes | 57/794 | 0.072 (0.056-0.092) | 0.463 |
|  |  | No | 18/209 | 0.086 (0.055-0.132) |  |
|  | Dominant Approach | Bilateral | 11/192 | 0.057 (0.032-0.100) | **0.018*** |
|  |  | Unilateral | 9/58 | 0.155 (0.084-0.269) |  |
| **Anisocoria** | Country | Non-US | 2/40 | 0.050 (0.014-0.165) | 0.999 |
|  |  | US | 2/49 | 0.041 (0.011-0.137) |  |
|  | Study Type | Observational | 2/68 | 0.029 (0.008-0.101) | 0.235 |
|  |  | Interventional | 2/21 | 0.095 (0.027-0.289) |  |
|  | Study Design | Retrospective | 2/68 | 0.029 (0.008-0.101) | 0.235 |
|  |  | Prospective | 2/21 | 0.095 (0.027-0.289) |  |
|  | Sample Size | >30 | 2/37 | 0.054 (0.015-0.177) | 0.999 |
|  |  | <30 | 2/52 | 0.038 (0.011-0.130) |  |
|  | Muscle Disinsertion | Yes | 0/12 | 0.000 (0.000-0.242) | 1.000 |
|  |  | No | 4/77 | 0.052 (0.020-0.126) |  |
|  | Dominant Approach | Bilateral | 0/19 | 0.000 (0.000-0.168) | 0.327 |
|  |  | Unilateral | 4/58 | 0.069 (0.027-0.164) |  |

***Note:*** *ONSF= Optic Nerve Sheath Fenestration; CI = Confidence Interval; NA = Not Available; P-values represent comparison between subgroups within each category; US= United States; * Denotes Statistical Significance.*

## **Supplementary Table 4: Publication Bias Assessment of Outcomes.**

| Outcome | Number of Studies | Egger's Test (p-value) | Funnel Plot Asymmetry | Trim-and-Fill (Missing Studies) | Adjusted Effect Size (95% CI) | Risk of Publication Bias |
| --- | --- | --- | --- | --- | --- | --- |
| Visual Acuity Improvement | 19 | 0.034 | Present (Right-skewed) | 4 | 0.312 (0.284-0.341) [Original: 0.345 (0.318-0.373)] | Suspected (Moderate) |
| Visual Field Improvement | 16 | 0.245 | Symmetric | 2 | 0.682 (0.645-0.718) [Original: 0.694 (0.659-0.727)] | Low |
| Optic Disc Resolution | 11 | 0.789 | Symmetric | 0 | 0.909 (0.874-0.935) [No adjustment needed] | Low |

**Notes:**
1. Egger's test p-value < 0.05 indicates statistically significant publication bias.
2. Funnel plot asymmetry assessment based on visual inspection and statistical testing.
3. Trim-and-fill method estimates the number of missing studies and adjusts the effect size accordingly.
4. Risk of publication bias categorization:
 • ***Low:*** No evidence of asymmetry, non-significant Egger's test.
 • ***Moderate:*** Some evidence of asymmetry or significant Egger's test.
 • ***High:*** Strong evidence of asymmetry with significant impact on effect size.

**Methods:**
• Egger's regression test performed using standard methods.
• Funnel plot asymmetry assessed using both visual inspection and statistical methods.
• Trim-and-fill analysis conducted using random-effects model.
• Adjusted effect sizes calculated simulated effect of missing studies.
• Risk of publication bias assessed using multiple criteria including statistical tests and visual assessment.

**Statistical Analysis:**
• Significance level set at p < 0.05 for Egger's test.
• Random-effects model used for all analyses.
